# Circulating ECV-Associated miRNAs as Potential Clinical Biomarkers in Early Stage HBV and HCV Induced Liver Fibrosis

**DOI:** 10.3389/fphar.2017.00056

**Published:** 2017-02-09

**Authors:** Joeri Lambrecht, Pieter Jan Poortmans, Stefaan Verhulst, Hendrik Reynaert, Inge Mannaerts, Leo A. van Grunsven

**Affiliations:** ^1^Liver Cell Biology Lab, Department of Basic Biomedical Sciences, Vrije Universiteit BrusselBrussels, Belgium; ^2^Department of Gastroenterology and Hepatology, Universitair Ziekenhuis BrusselBrussels, Belgium

**Keywords:** hepatic stellate cell, extracellular vesicles, chronic liver disease, plasma, non-coding RNA

## Abstract

**Introduction:** Chronic hepatitis B (HBV) and C (HCV) virus infection is associated with the activation of hepatic stellate cells (HSCs) toward a myofibroblastic phenotype, resulting in excessive deposition of extracellular matrix, the development of liver fibrosis, and its progression toward cirrhosis. The gold standard for the detection and staging of liver fibrosis remains the liver biopsy, which is, however, associated with some mild and severe drawbacks. Other non-invasive techniques evade these drawbacks, but lack inter-stage specificity and are unable to detect early stages of fibrosis. We investigated whether circulating vesicle-associated miRNAs can be used in the diagnosis and staging of liver fibrosis in HBV and HCV patients.

**Methods**: Plasma samples were obtained from 14 healthy individuals and 39 early stage fibrotic patients (F0–F2) with chronic HBV or HCV infection who underwent transient elastography (Fibroscan). Extracellular vesicles were extracted from the plasma and the level of miRNA-122, -150, -192, -21, -200b, and -92a was analyzed by qRT-PCR in total plasma and circulating vesicles. Finally, these same miRNAs were also quantified in vesicles extracted from *in vitro* activating primary HSCs.

**Results**: In total plasma samples, only miRNA-200b (HBV: *p* = 0.0384; HCV: *p* = 0.0069) and miRNA-122 (HBV: *p <* 0.0001; HCV: *p* = 0.0007) were significantly up-regulated during early fibrosis. In circulating vesicles, miRNA-192 (HBV: *p <* 0.0001; HCV: *p <* 0.0001), -200b (HBV: *p <* 0.0001; HCV: *p <* 0.0001), -92a (HBV: *p <* 0.0001; HCV: *p <* 0.0001), and -150 (HBV: *p* = 0.0016; HCV: *p* = 0.004) displayed a significant down-regulation in both HBV and HCV patients. MiRNA expression profiles in vesicles isolated from *in vitro* activating primary mouse HSCs resembled the miRNA expression profile in circulating vesicles.

**Conclusion**: Our analysis revealed a distinct miRNA expression pattern in total plasma and its circulating vesicles. The expression profile of miRNAs in circulating vesicles of fibrotic patients suggests the potential use of these vesicle-associated miRNAs as markers for early stages of liver fibrosis.

## Introduction

Liver fibrosis is the pathological condition of the liver resulting from sustained wound healing in response to various causes of chronic liver injury, including chronic hepatitis B virus (HBV) and hepatitis C virus (HCV) infection (Friedman, 2003; Wallace et al., 2008). The main collagen producing cell type in this pathological process is the hepatic stellate cell (HSC), which is a liver-resident cell, located in the Space of Disse, that can activate toward a myofibroblastic (fibrotic) state during chronic liver injury (Hautekeete and Geerts, 1997). This activation process is associated with major changes in the gene-expression profile of the stellate cell (Mann and Smart, 2002; De Minicis et al., 2007), with as most important consequence, a dysregulation of the production of extracellular-matrix proteins, and thus excessive deposition and accumulation of extracellular matrix (ECM) in the parenchyma of the liver (Li and Friedman, 1999). Liver fibrosis can progress to a cirrhosis, characterized by a loss of liver function, and even hepatocellular carcinoma (HCC) (Schuppan and Afdhal, 2008). Liver disease remains a global burden, as demonstrated by a study of the Global Burden of Disease (GBD) project, which estimated in 2010 a death toll of over one million deaths due to liver cirrhosis, and an additional one million deaths due to liver cancer and acute hepatitis (Lozano et al., 2012; Byass, 2014).

The diagnosis of liver fibrosis can be made by routine examinations, but the gold standard remains the liver biopsy, which identifies the underlying cause, assesses the necro-inflammatory grade and identifies the stage of fibrosis (Friedman, 2003). This diagnostic procedure is however, invasive and is associated with a small risk of pain, and very rarely bleeding or other severe complications (Thampanitchawong and Piratvisuth, 1999; Bravo et al., 2001). In addition, due to inter-observer variability and sample errors, there is a possibility of under- or over-rating the specific stage of liver fibrosis (Maharaj et al., 1986; Regev et al., 2002). Because of these drawbacks, researchers use several non-invasive techniques to measure the degree of liver fibrosis, including scoring systems evaluating biochemical parameters such as the Fibrotest score (Imbert-Bismut et al., 2001) and the aspartate transaminase to platelet ratio index (APRI) test (Wai et al., 2003), and imaging techniques such as transient elastography (Fibroscan) (Sandrin et al., 2003; Foucher et al., 2006). Although many of the non-invasive techniques have a significant predictive value for the diagnosis of later stages of liver fibrosis, being significant liver fibrosis and liver cirrhosis, many of these systems have not been validated for the identification of early stages of disease progression or to discriminate between the various stages (Adams, 2011; Sharma et al., 2014).

One potential new diagnostic method for the identification and follow up of liver fibrosis progression could be the analysis of circulating miRNAs. MiRNAs are short RNA sequences of ∼22 nucleotides that regulate gene expression post-transcriptionally and can be found both in the intra- and extracellular environment (Bartel and Chen, 2004; Lee, 2013). MiRNAs obtain extracellular stability thanks to binding with lipids, proteins, and packaging into extracellular vesicles (ECVs) (Turchinovich et al., 2012). This latter group consists of small membrane-derived vesicles, of which 2 subtypes can be found in the blood stream: larger (100 nm-1 μm in diameter) vesicles, also known as microvesicles or shedding vesicles (Camussi et al., 2010), and smaller vesicles (30–100 nm in diameter), also known as exosomes (Thery et al., 2002). Interestingly, an increased number of circulating vesicles has been shown in mice and humans with alcoholic hepatitis (Momen-Heravi et al., 2015) and in murine models of NAFLD (Povero et al., 2014). In addition, communication experiments have proven the functionality of miRNA-baring vesicles in various aspects of the liver fibrosis process, such as HSC activation status (Charrier et al., 2014; Wang R. et al., 2015), and angiogenesis (Witek et al., 2009; Povero et al., 2013). Their increased presence during liver fibrosis and their importance in cell communication makes circulating ECVs potential candidates to act as biomarkers for liver fibrosis.

Based on the major ECM-producing character of activating HSCs during the process of liver fibrosis (Hautekeete and Geerts, 1997; Atzori et al., 2009), we hypothesized that HSCs contribute to this increase in circulating ECVs. In this study, we compared total circulating miRNAs with ECV-associated miRNAs for their ability to be used as a diagnostic tool for early stage liver fibrosis caused by chronic HBV and HCV infection. After identification of miRNAs that are differentially regulated during the process of HSC activation, we analyzed which group of circulating miRNAs could better represent this changing HSC status. Our data shows that the selected miRNAs do not display the same expression patterns in ECVs and total plasma during early stage liver fibrosis by chronic HBV and HCV infection, and that ECV-associated miRNAs display a tendency of representing the changing cellular miRNA content during HSC activation.

## Materials and Methods

### Isolation and Culture of Mouse HSCs

The university’s guidelines for the care and use of laboratory animals in research were strictly followed and the study was approved by the ethical committee of the Vrije Universiteit Brussel in project 15-212-1. Quiescent HSCs were isolated from male Balb/c mice (Charles River Laboratories, L’Arbresle, France) (25–30 weeks old) as described earlier (Mannaerts et al., 2010). After isolation, mouse HSCs were cultured in Dulbecco’s modified Eagle’s medium (Lonza, Verviers, Belgium) supplemented with 10% Exosome-depleted fetal bovine serum (System Biosciences, Mountain View, USA), 2 Mm L-glutamine (Ultraglutamine 1^®^) (Lonza), 100 U/ml penicillin and 100 μg/ml streptomycin (Pen-Strep^®^) (Lonza).

### Patient Population

Between October 2015 and July 2016 39 patients were recruited. Patients presented at the department of Gastro-enterology and Hepatology of the UZ Brussel with chronic liver disease caused by Hepatits B or C infection underwent a Fibroscan^®^ (Echosens, Paris, France). None of the patients was under treatment for HBV or HCV infection. Patients who were pregnant, or had a concomitant disease that could possibly bias the results (such as HIV, alcohol abuse) were excluded. A healthy population of 14 individuals was recruited as a control group. In this healthy population, liver function was not tested, but none of the subjects had a history of liver disease, and all subjects were declared healthy after the annual exam by the occupational physician. Characteristics of the study cohort are listed in **Table 1**. The study was performed according to the principles of the declaration of Helsinki, and approved by the ethical committee of the UZ Brussel and Vrije Universiteit Brussel (reference number 2015/297). Written informed consent was obtained from every participant before enrolment.

**Table 1:** Baseline characteristics of included patients.

	HBV-infected	HCV-infected	Healthy
Individuals (n)	19	20	14
Male/Female (n)	11/8	8/12	6/8
Age (years)	43.00 ± 13.76	54.30 ± 16.85	36.62 ± 15.10
BMI (kg/m^2^)	26.56 (24.08–29.03)	25.83 (22.52–27.48)	
**Scoring systems**			
Liver stiffness (kPa)	4.500 (3.800–6.100)	5.900 (3.900–7.100)	
FIB-4 index	1.230 (0.6750–2.308)	1.900 (1.050–2.840)	
**Blood parameters**			
ALT (IU/L)	40.00 (25.50–59.25)	40.50 (25.75–67.75)	
AST (IU/L)	31.50 (24.00–49.25)	41.50 (27.00–65.00)	
Alkaline phosphatase (IU/L)	64.00 (47.25–71.50)	66.00 (51.50–85.00)	
GGT (IU/L)	22.00 (17.25-37.75)	43.00 (19.50-135.50)	
Total bilirubine (mg/dL)	0.5450 (0.4375–0.8850)	0.6050 (0.3800–0.8425)	
Albumin (g/L)	44.50 (42.25–45.75)	43.00 (41.00–46.00)	
INR	1.100 (1.000–1.100)	1.000 (1.000–1.150)	
Alfa-fetoprotein (kIU/L)	2.900 (2.300–3.800)	3.850 (2.800–5.800)	
Platelet count (10^3^/mm^3^)	179.5 (148.8–220.5)	249 (205.0–321.0)	
Serum creatinine (mg/dL)	0.9050 (0.7925–0.9450)	0.9400 (0.6700–1.040)	


*Ages are given as means ± SD, other parameters are givens as medians (IQR). BMI, body mass index; HBV, hepatitis B virus; HCV, hepatitis C virus; FIB-4, fibrosis-4; AST, aspartate aminotransferase; ALT, alanine aminotransferase; GGT, gamma-glutamyl transpeptidase; INR, international normalized ratio.*

### Assessment of Liver Fibrosis

Staging of liver fibrosis was assessed by Fibroscan (Echosens, Paris, France), a device based on transient elastography. Only patients with at least 10 valid liver stiffness measurements, with an interquartile range of less than 30% of the median liver stiffness, and with a success rate of at least 60% were included in the study. Patients who presented with a liver stiffness below 9.5 kPa were considered having no significant or moderate liver fibrosis (F0-F1-F2). This cut-off value is based on current literature, and can be used for both patients infected with HBV as with HCV (Wang et al., 2009). No patients with liver stiffness of 9.5 kPa or more (corresponding to advanced liver fibrosis or liver cirrhosis) were included in the study population.

### Plasma Collection and Storage

Blood samples were collected by venepuncture into Sarstedt *S*-monovette EDTA 9ml tubes (Sarstedt AG & Co, Nümbrecht, Germany) at the department of Gastro-enterology and Hepatology of the UZ Brussel. Samples were kept on ice and were processed within 1 h of collection by a two-step centrifugation process consisting of 1500 g for 10 min followed by 2000 g for 3 min to eliminate any remnant cells. The supernatant was transferred to RNase/DNase-free tubes and stored at -80°C until further use.

### Plasma miRNA Analysis

Five hundred microliter of plasma was used. Plasma miRNA was extracted using the Nucleospin^®^ miRNA Plasma kit (Macherey-Nagel, Düren, Germany) using the manufacturers protocol. Caenorhabditis elegans miRNA-39 (cel-miR-39) (Qiagen, Hilden, Germany) was spiked into the plasma lysate before extraction, and served as an external processing control.

### Vesicle Isolation and Analysis from Plasma and Cell Culture Medium

Five hundred microliter of plasma was mixed with Total Exosome Isolation (TEI) reagent (Thermo Fisher scientific, Waltham, MA, USA), and isolation was performed according to the manufacturer’s protocol. In summary, the plasma sample was depleted of debris by a two-step centrifugation of 20 min at 2000 and 10000 *g*. After incubating the plasma sample for 10 min at room temperature with the reagent, vesicles were precipitated by centrifugation at 10000 *g*, and resuspended in a volume of PBS. For the isolation of vesicles from culture medium (CM), 4 ml of CM was centrifuged for 30 min at 2000 *g*, to remove any contaminating debris, followed by overnight incubation with the TEI precipitation reagent, and centrifugation for 1 h at 10000 *g* to generate a vesicle-rich pellet. The obtained vesicle suspensions from plasma and CM were purged from contaminating proteins by incubation with 0.5 mg proteinase K (Thermo Fisher scientific) for 30 min at 55°C. Total RNA was extracted from these vesicles by use of the Quick RNA miniprep (Zymo Research, Irvine, CA, USA). Synthetic spike-in cel-miR-39 was added to the vesicle lysate before proceeding with the manufacturer’s protocol.

### Quantitative Real-Time Polymerase Chain Reaction

Both total RNA obtained from the vesicle fraction, and miRNAs obtained from the plasma were reverse transcribed to cDNA by use of miScript II RT kit (Qiagen, Hilden, Germany). Total cell RNA was extracted and purified using the Reliaprep RNA cell Miniprep System (Promega, Madison, WI, USA) and converted to cDNA by reverse transcription using the Revert Aid Kit (ThermoFisher Scientific, St. Leon-Rot, Germany). The expression profiles of selected mRNA/miRNA were analyzed by the ABI 7500 Real time PCR system (Applied Biosystems, Waltham, MA, USA) using the SYBR green PCR method. MiRNA detection was established by use of the miScript Universal primer in combination with miRNA-specific primers, listed in **Table 2**. Primers used for detection of mRNA are listed in **Table 3**. The quantitative value for a given miRNA and mRNA was obtained by subtracting the cycle threshold of the internal reference respectively cel-miR-39 and *Gapdh*, with the cycle threshold of the analyzed miRNA or mRNA.

**Table 2:** miRNA-specific primers.

miRNA	Mature miRNA primer
miR-92a-3p	TATTGCACTTGTCCCGGCCTGT
miR-122-5p	TGGAGTGTGACAATGGTGTTTG
miR-150-5p	TCTCCCAACCCTTGTACCAGTG
miR-192-5p	CTGACCTATGAATTGACAGCC
miR-200b-3p	TAATACTGCCTGGTAATGATGA
miR-21-5p	TAGCTTATCAGACTGATGTTGA
cel-miR-39-3p	AGCTGATTTCGTCTTGGTAATA


*All primers were ordered from Integrated DNA Technologies (IDT, Leuven, Belgium), and are specific for detection of both human and murine miRNA-expression. Lyophilized primers were dissolved in RNase-free water to obtain primer solutions with a final concentration of 10 pmol/μl.*

**Table 3:** Messenger RNA (mRNA) primers.

Gene	Messenger RNA (mRNA) primer
*Gapdh*	FP: TCGAGATCGCCACCTACAG
	RP: GTCTGTACAGGAATGGTGATGC
*Lox*	FP: CTCCTGGGAGTGGCACAG
	RP: CTTGCTTTGTGGCCTTCAG
*Col1a1*	FP: ACCTAAGGGTACCGCTGGA
	RP: ACCTAAGGGTACCGCTGGA
*Acta2*	FP: CCAGCACCATGAAGATCAAG
	RP: TGGAAGGTAGACAGCGAAGC


*All primers were ordered from Integrated DNA Technologies (IDT, Leuven, Belgium), and are specific for detection of murine mRNA-expression. Lyophilized primers were dissolved in RNase-free water to obtain primer solutions with a final concentration of 10 pmol/μl. FP, forward primer; RP, reverse primer.*

### Protein Analysis and Western Blot

The vesicle pellet obtained by use of the Total exosome isolation (TEI) reagent was lysed in 50 μl of RIPA buffer supplemented with complete protease-inhibitors (Roche Diagnostics, Mannheim, Germany) and PhosSTOP phosphatase-inhibitors (Roche Diagnostics). Lysates were sonicated twice (15 s) in a 4°C water bath. Protein concentrations were quantified using the Micro BCA^TM^ Protein assay kit (Thermo Fisher Scientific) according to manufacturer’s instructions. Twenty five microgram of vesicles extracted from plasma or CM were for western blotting. Protein expression was assessed using antibodies against CD63, TSG101 (both 1:200, Santa Cruz Biotechnology, Dallas, TX, USA), β-actin, vimentin (1:5000 and 1:500 respectively, Sigma–Aldrich, St.Louis, MO, USA), collagen type I (1:1000, Abcam, Cambridge, UK), and PDGFRβ (1:1000, GeneTex, Irvine, CA, USA). Calreticulin (1:1000, Cell Signaling Technology, Danvers, MA, USA) was used to verify absence of cell contamination. As positive control, a protein cell lysate of the HepG2 cell line was used.

### Statistical Analysis

The statistical analysis was performed using the GraphPad Prism 6 (GraphPad, Palo Alto, USA) statistical software. Results are given as means ± standard deviation (SD). Analysis of variance (ANOVA), followed by Tukey’s post-test was employed for statistical analysis. The sufficiency of the sample size was confirmed by a web-based calculator^1^, using in house preliminary results and a type I error rate (α) of 5% and a power (1-β) of 80%. Receiver operating characteristic (ROC) curves were constructed, and the area under the curve (AUC) was calculated as accuracy index for the diagnostic performance of the selected miRNAs. Results were considered statistically significant when *P <* 0.05.

## Results

### Clinical Characteristics of the Patient Population

A cohort of 39 patients with early liver fibrosis was enrolled into this study, including 19 patients with chronic HBV infection, and 20 patients with chronic HCV infection. All patients underwent transient elastography (Fibroscan) to exclude advanced stage liver fibrosis or cirrhosis. Fourteen healthy individuals with absence of liver disease symptoms or history of liver disease were included as control population. Participant characteristics and biochemical parameters are depicted in **Table 1**. The two patient populations displayed no significant difference in Fibroscan-determined liver stiffness. The platelet count was slightly higher in the HCV infected population (*p* = 0.0317), but no other parameters showed any significant difference. The values of all biochemical parameters were in the normal reference range, further indicating the absence of any severe fibrosis or cirrhosis.

### Identification of Deregulated miRNAs in Activated Human HSCs and in the Circulation of Fibrotic Patients

To identify circulating miRNAs that might represent early changes in the fibrotic liver, we turned to HSC activation, a key process in this pathology. Upon chronic liver damage by HBV or HCV infection, HSCs undergo an activation process toward a myofibroblastic phenotype, which is associated with dynamic changes in gene and miRNA expression profiles (De Minicis et al., 2007; Coll et al., 2015). We hypothesized that those deregulated miRNAs are secreted into the blood stream and their blood levels might serve as markers for fibrosis progression. We probed the literature for deregulated miRNAs previously identified in activated human HSCs (Coll et al., 2015), for their prevalence in the blood stream during liver disease (**Table 4**). MiRNA-150, -192, and -200b were selected from this list as potentially important miRNAs, based on their significant deregulation during HSC activation and their changing expression in the blood of patients with liver disease. MiRNA-122 was used as positive control, as its changing expression in circulation has already been widely described for various causes of liver fibrosis, including HBV (Xu et al., 2011; Waidmann et al., 2012; Winther et al., 2013) and HCV (Cermelli et al., 2011; Wang J.H. et al., 2015; Motawi et al., 2016) and is strongly correlated with hepatocyte damage (Farid et al., 2012; Roderburg et al., 2015). MiRNA-92a and miRNA-21 were selected for their known presence in circulating vesicles from patients with various pathologies (Skog et al., 2008; Taylor and Gercel-Taylor, 2008; Rabinowits et al., 2009; Chen et al., 2016), and their potential implication in liver disease.

**Table 4:** *In silico* analysis of miRNAs differentially regulated during activation of human HSCs (Coll et al., 2015) and in the circulation of patients with liver disease.

	Change of expression in human aHSC (Fold Change)	Expression in plasma of liver disease-compared to healthy individuals		Source
			
		Expression trend	Etiology	
miRNA-150-5p	↓ (870)	↑	HBV	Li et al., 2010; Venugopal et al., 2010
miRNA-192-5p	↓ (8.53)	↑	HBV, NASH, NAFLD	Tryndyak et al., 2012; Winther et al., 2013; Becker et al., 2015; Pirola et al., 2015
miRNA-200b-3p	↑ (22.55)	↑	NAFLD, HBV/HCV-associated HCC	Murakami et al., 2011; Tryndyak et al., 2012
miRNA-122-5p	N.D.	↑	HCV, HBV, NASH, NAFLD	Arataki et al., 2013; Shrivastava et al., 2013; Tan et al., 2014; Pirola et al., 2015
miRNA-21-5p	N.D.	= /↑	HBV, NAFLD	Yamada et al., 2006; Cermelli et al., 2011; Becker et al., 2015
miRNA-92a-3p	N.D.	↑	HBV-associated HCC, HCV	Li et al., 2010; Ji et al., 2011; Shrivastava et al., 2013


*N.D., non-determined.*

### MiRNA-122, -192, and -200b Are Up-Regulated in Total Plasma of Early Stage Fibrotic Patients

**FIGURE 1:**
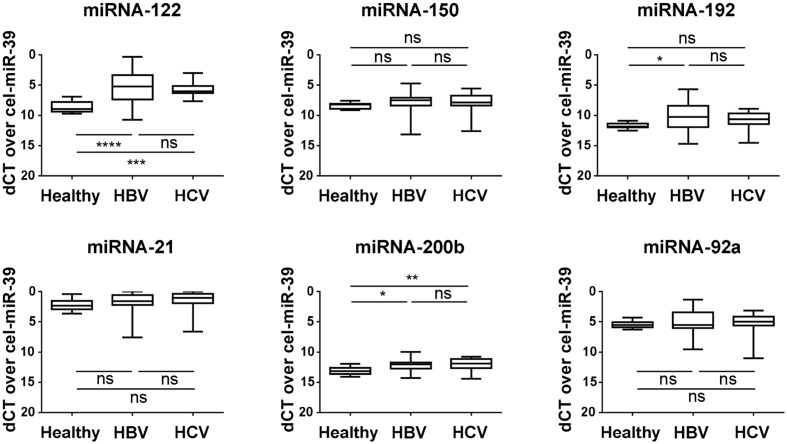
**Plasma miRNAs are differentially regulated during early fibrosis in patients with chronic HBV and HCV infection.** The plasma levels of miRNA-122, -150, -192, -21, -200b, and -92a were measured by real-time quantitative PCR, and obtained Ct levels were normalized by use of spiked-in cel-miRNA-39. The miRNA expression levels in plasma of F0/F1/F2 patients with chronic HBV and HCV infection were compared with healthy control subjects. ^∗^*P <* 0.05; ^∗∗^*P <* 0.01; ^∗∗∗^*P <* 0.001; ^∗∗∗∗^*P <* 0.0001.

The expression of selected miRNAs was first analyzed in total plasma samples from early stage chronic HBV- and HCV-infected fibrotic patients, and compared with the healthy control group (**Figure 1**). Significantly higher expression of miRNA-122 in the patient groups confirms earlier reports by Trebicka et al. (2013) and Arataki et al. (2013), which showed that higher miRNA-122 levels reflect early stages of chronic HCV and HBV induced liver fibrosis. From our selected miRNAs, miRNA-21, -92a, and -150 displayed no significant change in expression. MiRNA-192 was significantly up-regulated in HBV-patients, but not in HCV patients, when compared with the control group. MiRNA-200b, was significantly up-regulated in both HBV and HCV fibrotic patients.

### Early Stage Fibrotic Patients Have an Increased Circulating ECV-Content With Low Levels of miRNA-192, -92a, -200b, and -150

**FIGURE 2:**
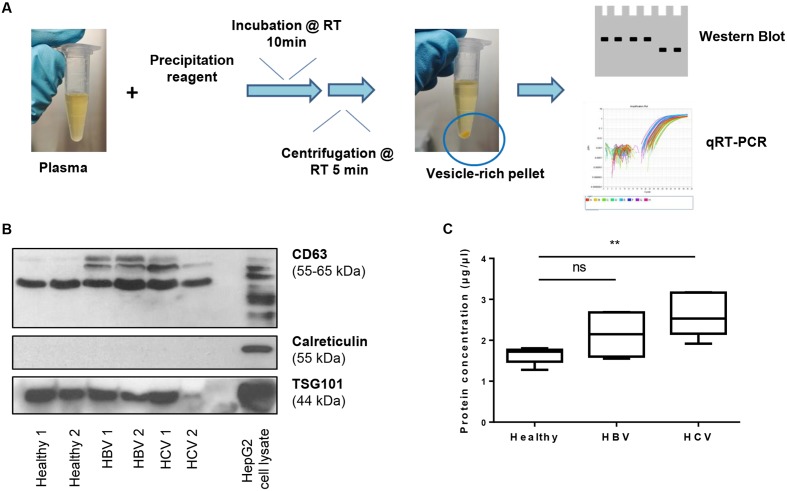
**Isolation of extracellular vesicles from plasma.** **(A)** ECVs were extracted from 0.5 ml plasma obtained from F0/F1/F2 patients with chronic HBV and HCV infection, and a healthy control group. After centrifugation, a vesicle-rich pellet is obtained, which is dissolved in PBS for further analysis. **(B)** Obtained vesicle-suspensions were analyzed by western blot for expression of the vesicle-markers CD63 and TSG101, and the absence of the cellular marker calreticulin. **(C)** Protein content of ECVs isolated from different groups. An increase in protein concentration can be seen comparing vesicle-preparations of healthy subjects with HBV and HCV patients. ^∗∗^*P* < 0.01.

Next, we analyzed the expression of the selected miRNAs in vesicles isolated from plasma from healthy controls and patients with HBV or HCV infection, using standardized precipitation protocols (Schageman et al., 2013; Li et al., 2015) (**Figure 2A**). Western blot analysis for the vesicle-enriched proteins CD63 and TSG101 (Yoshioka et al., 2013; Kowal et al., 2016) demonstrates the presence of vesicles in the preparations, while the cellular protein calreticulin (Jeppesen et al., 2014) was absent (**Figure 2B**). The expression of CD63, a tetraspanin family member, can be seen as multiple bands for HCV and HBV patients, while healthy subjects have a single band. This suggests a potential disease-related presence of CD63 isoforms on the vesicle membrane. All vesicle samples were negative for calreticulin, indicating the absence of ‘contaminating’ cells in the vesicle suspension. As an increase of circulating vesicles has been proven during the process of alcoholic hepatitis (Momen-Heravi et al., 2015) and murine models of non-alcoholic fatty liver disease (NAFLD) (Povero et al., 2014), we also determined their relative presence in early fibrotic HBV and HCV patients, compared to the healthy control subjects. A significant enrichment of circulating vesicle-content was found in HCV fibrotic patients, while a trend of such enrichment was found in the HBV group (**Figure 2C**). As both patient-groups have an equal distribution in liver stiffness, as assessed by Fibroscan, and biochemical characteristics (**Table 1**), we suggest that the level of enrichment of circulating ECVs is disease-dependent.

**FIGURE 3:**
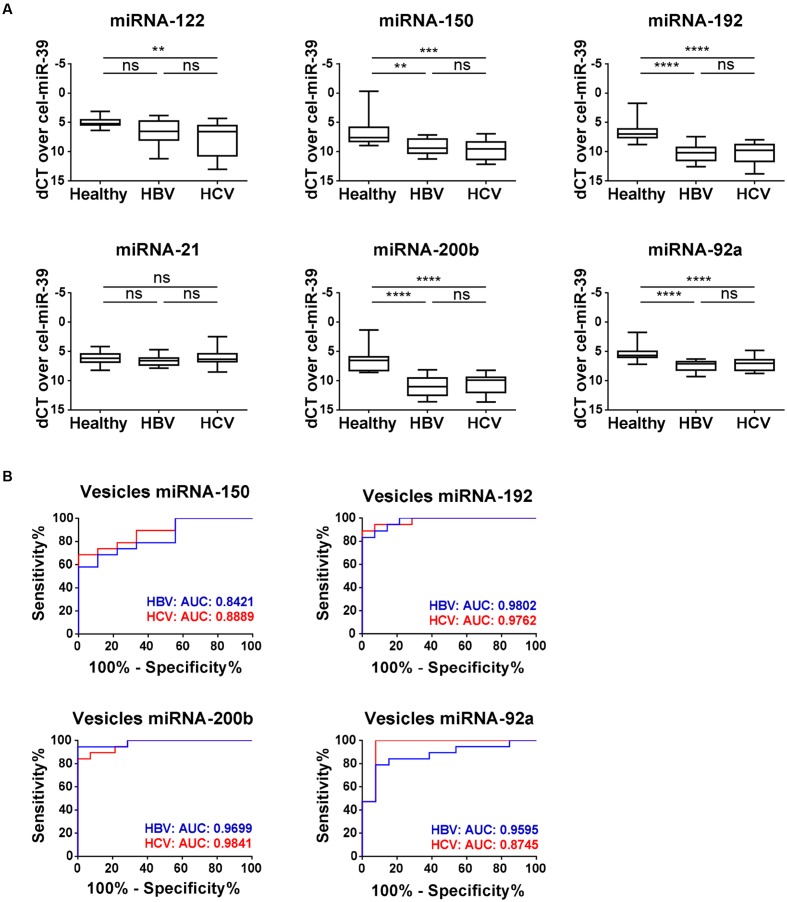
**Expression of vesicle-associated miRNA-150, -192, -200b, -92a during early stage HBV- and HCV-induced fibrosis.** **(A)** The levels of plasma vesicle-associated miRNA-122, -150, -192, -21, -200b, and -92a were measured by real-time quantitative PCR, and obtained Ct levels were normalized by use of spiked-in cel-miRNA-39. **(B)** Curves of receiver operating characteristic (ROC) analysis using the differentially expressed vesicle-associated miRNA-150, -192, -200b, and -92a for discriminating early stage HBV (blue) and HCV (red) fibrotic patients versus healthy controls. ^∗∗^*P <* 0.01; ^∗∗∗^*P <* 0.001; ^∗∗∗∗^*P <* 0.0001.

Analysis of the vesicle samples for their miRNA cargo showed that the expression profile of the selected miRNAs was different from their profile in total plasma samples (**Figure 3A**). While miRNA-122 levels were significantly up-regulated in total plasma of both HBV and HCV fibrotic patients, we observed a significant down-regulation of miRNA-122 in vesicles of the HCV population, and no change in HBV samples. MiRNA-21 shows, just as in the total plasma samples, no change in expression in the vesicle preparations. A significant down-regulation in vesicle-associated miRNA expression in both patient groups could be seen for miRNA-192, -150, -92a, and -200b. Analysis of these four latter miRNAs with use of ROC plots identifies for all miRNAs a strong discriminative potential between the healthy and fibrotic HBV and HCV patient groups (**Figure 3B**). Especially miRNA-192 (HBV AUC: 0.9802; HCV AUC: 0.9762) and miRNA-200b (HBV AUC: 0.9699; HCV AUC: 0.9841) have an inherent potential to be used as biomarker for the identification of early fibrosis by chronic HBV and HCV infection. MiRNA-92a shows a significant discriminative potential for HBV fibrotic patients (AUC: 0.9595), while this is less for HCV fibrotic subjects (AUC: 0.8745).

### Expression Patterns of miRNA-192, -92a, and -200b Show an Overlap in Circulating Vesicles and Activating HSCs

**FIGURE 4:**
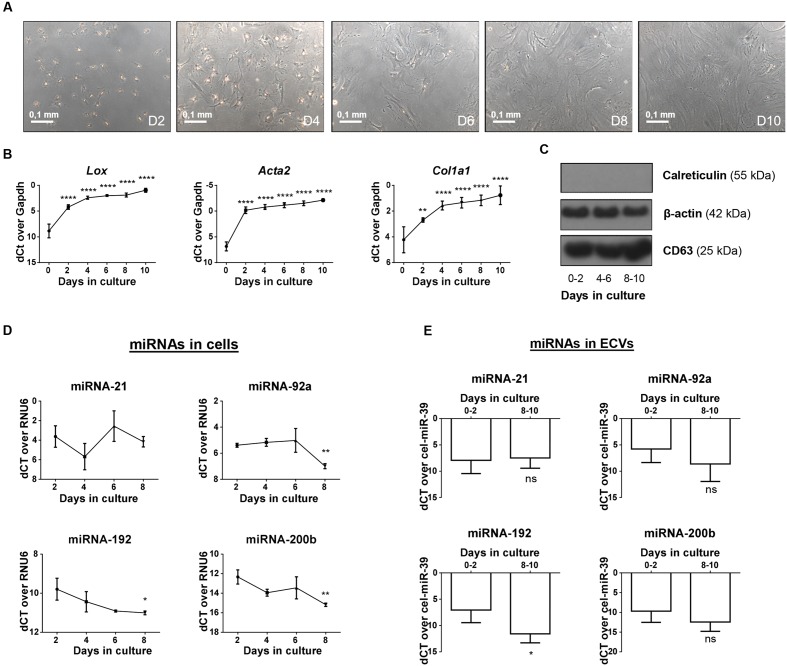
***In vitro* activating mouse HSCs; cellular and vesicle miRNA content.** Culture of primary mouse HSCs on regular tissue culture plates leads to the spontaneous initiation of the activation process toward a myofibroblastic phenotype, as can be seen by **(A)** a change in morphology of the cells and **(B)** the upregulation of the activation-associated markers *Acta2. Lox*, and *Col1A1*. **(C)** Vesicles isolated at different time points of the culture were verified for the presence of CD63 and beta-actin, and the absence of the negative marker calreticulin. Expression of miRNA-21, -92a, -192, and -200b was analyzed both in **(D)** cell lysates and **(E)** shedded vesicles extracted from *in vitro* activating HSCs by real-time quantitative PCR. Expression is calculated relative to the quiescent (day 2) time point. ^∗^*P <* 0.05; ^∗∗^*P <* 0.01; ^∗∗∗∗^*P <* 0.0001.

To determine whether the expression profile of miRNAs analyzed in the ECV fractions could reflect the expression profile of these miRNAs in activating HSCs, we investigated whether these miRNAs are present in vesicles released from activating HSCs. Hereto we collected vesicles from primary culture-activated mouse HSCs and investigated their miRNA content. First, HSC activation over a 10 days period was confirmed by the change in morphology of the HSCs (**Figure 4A**) and the increasing expression of the activation markers *Acta2, Col1a1*, and *Lox* (**Figure 4B**). During this activation process, the cellular expression of the 3 miRNAs that were identified as potential vesicle-associated fibrosis markers (miRNA -192, -200b, and -92a) and a control miRNA (miRNA-21) were analyzed (**Figure 4D**). MiRNA-21 shows a relatively stable expression, while levels of miRNA-192, -92a, and -200b are all down-regulated in activated HSCs. Vesicles extracted from the culture medium were characterized by western blot for the presence of CD63 and the absence of calreticulin (**Figure 4C**). The miRNA content of these vesicles displayed overlap with their cellular expression, as miRNA-21 was stable, miRNA-192 was significantly down-regulated, and miRNA-92a, and -200b show clear trends of being down-regulated during HSC activation (**Figure 4E**).

## Discussion

Liver fibrosis and cirrhosis are slowly progressing consequences of chronic liver damage and the incredible regenerative capacity of the liver impedes the early detection of fibrosis. Over the years, many have tried to develop sensitive, non-invasive methods for detection of liver fibrosis and cirrhosis. While the potential of circulating miRNAs is becoming clearer, most studies used cohorts of severe fibrotic and cirrhotic patients (Cermelli et al., 2011; Bala et al., 2012; Chen et al., 2013; Roderburg and Luedde, 2014). In this manuscript we investigated HSC-related miRNAs in ECVs isolated from early stage HBV and HCV plasma samples. We show that ECV-associated miRNA levels change in these patients when compared with healthy control plasma and we suggest that ECV-associated miRNAs can represent the activation status of the scar producing HSC.

Circulating vesicles have been explored extensively as diagnostic tools for cancer (Duijvesz et al., 2011; Cazzoli et al., 2013; Ogata-Kawata et al., 2014; Thakur et al., 2014). For example, Sohn et al. (2015) analyzed the expression of ECV-associated miRNAs between a cirrhotic population, patients with hepatocellular carcinoma (HCC), and a chronic HBV-infected control group. They suggest that total plasma miRNAs in comparison with ECV-associated miRNAs were less suitable to discriminate between HCC and HBV (Sohn et al., 2015). In accordance with these observations we describe significantly lower levels of miRNAs-192, -92, and -200b in hepatitis ECVs, while they have no diagnostic value when analyzed in total plasma.

Because HSCs are the main cellular source of scar proteins, we hypothesized that HSCs could also be a source of ECV-associated miRNAs in the circulation of fibrotic patients. MiRNA-192 was significantly up-regulated in total plasma of early stage HBV patients (**Figure 1**), but it shows significant down-regulation in the ECVs extracted from both HBV- and HCV-infected patient populations when compared to healthy subjects (**Figure 3A**). Additionally, analysis of cell lysates and ECVs extracted from the CM of *in vitro* activating HSCs show the same significant decreasing levels of miRNA-192 (**Figures 4D,E**). In previous work, we showed that also *in vivo* activated mouse HSCs and total liver samples of cirrhotic patients with superimposed alcoholic hepatitis show such significant down-regulation of cellular miRNA-192. Over-expression of miRNA-192 leads to less cell proliferation and less PDGF-induced migration (Coll et al., 2015), suggesting that miRNA-192 might play a role in suppressing HSC activation. Analysis of potential targets of the down-regulated ECV-associated miRNAs by TargetScan (**Supplementary Table 1**), identified various genes involved in some fundamental processes of HSC activation, such as cell migration, cell proliferation, ECM organization, and signaling by transforming growth factor beta (TGFβ) (Reeves and Friedman, 2002; Friedman, 2003). This underlines the importance of the selected miRNAs in the activation process of the HSCs, and suggests a potential transmission of cellular status when transmitted to nearby HSCs by ECV communication, a mechanism that has already been studied *in vitro* (Chen et al., 2014).

We acknowledge that based on our ECV preparation we cannot really determine the contribution of HSCs to the circulating vesicle population. We did probe the obtained ECVs for presence of HSC-related markers (**Supplementary Figure 1**). Analysis of our vesicle populations for collagen type I (Lee et al., 1995) and platelet-derived growth factor receptor beta (PDGFRβ) (Borkham-Kamphorst et al., 2004; Thoen et al., 2011), showed an enhanced expression in the patient populations with early liver fibrosis, as compared to the healthy controls. Expression of vimentin, a fibroblast-associated intermediate filament known to have a constant expression during HSC activation (Van Rossen et al., 2014), was equally expressed in ECV populations of healthy and sick individuals. These selected markers are not unique for liver, but within the liver they are highly expressed in HSCs and suggest the possibility of HSC-derived ECVs to contribute to the total circulating ECV population. Ideally, HSC- specific ECVs should be isolated from the patient plasma samples. For this purpose, future studies could rely on the presence of PDGFRβ on ECVs for use in cell type-specific vesicle isolation strategies (Hong et al., 2014; Wang et al., 2016). Unfortunately, these methods are not widely used and still require optimization.

Of our analyzed miRNAs, miRNA-122 has been the most widely described and is considered a liver specific miRNA. Its expression in liver reduces upon injury, while significantly more miRNA-122 is detected in total plasma of fibrotic patients. Circulating miRNA-122 appears to mirror the histological and molecular events occurring in the liver and correlates with the degree of damage as measured by serum ALT levels. This correlation has been shown in advanced fibrotic NASH-patients (Pirola et al., 2015), NAFLD patients (Tan et al., 2014), and chronic HCV infected patients (Shrivastava et al., 2013). In this latter patient group, significant higher levels of circulating miRNA-122 could be seen in early stage (F0–F2) fibrosis, as compared to advanced fibrosis (F3–F4) (Trebicka et al., 2013). Also in our experiments, an enhanced presence of miRNA-122 could be found in the total plasma of early stage HBV- and HCV-infected patients. A potential explanation for this event could be the discharge of this miRNA by hepatocytes, as this cell type has inherent abundant levels of miRNA-122 (Pirola et al., 2015). During progression of liver fibrosis to cirrhosis, significant reduction of miRNA-122 can be seen in total liver tissue, which is reflected by the reduced circulating miRNA-122 levels during later stages of fibrosis (Trebicka et al., 2013) and potentially the result of less pronounced hepatocyte damage at these stages of disease. In our experiments, the levels of miRNA-122 were not affected in circulating ECVs from early fibrosis stage HBV and HCV patients. However, in the research of Bala et al. (2012), elevated levels of vesicle-associated miRNA-122 were found when comparing murine models of alcohol- and inflammation-induced liver disease with a control population. These findings were later confirmed in *in vitro*-experiments, where APAP-treated hepatocytes released ECVs that were rich in miRNA-122 (Holman et al., 2016). The extent of hepatocyte damage can of course differ between rather short term liver injury in rodents, *in vitro* induced hepatocyte toxicity, and chronic hepatitis infected patients. Together, our observations and the studies of Bala et al. (2012) and Holman et al. (2016) suggest that different settings of liver injury leading to hepatocyte death could be associated to ECV release of specific miRNAs.

Although our results suggest the possibility of these circulating vesicles to be used as part of a novel biomarker panel, we recognize that our study has limitations, such as the limited number of subjects included. However, even with this limited amount of subjects, significant changes could be found in the miRNA levels of total plasma and circulating ECVs. Analysis of larger cohorts of patients and of different etiologies will increase power and can perhaps provide the necessary evidence to include one or more of the proposed miRNAs in a future medical diagnostic panel for early stage liver fibrosis. Moreover, it could be interesting to include patient groups with late-stage (F3–F4) fibrosis to analyze their miRNA-associated ECV content. A second limitation is the absence of precise fibrosis staging of our included patients by liver biopsy. However, the use of transient elastography (Fibroscan) has already been extensively tested and validated for staging fibrosis in patients with viral hepatitis, and has been shown to be highly specific for excluding advanced fibrosis (Sterling et al., 2006; Afdhal et al., 2015). We have used this Fibroscan technique in combination with Fib-4 scoring, which has a negative predictive value of 90% for advanced liver fibrosis (Ishak stage 4–6), and which has been shown to be able to discriminate between fibrosis stage F0–2 and F3–4 (Verlinden et al., 2015; Zhang et al., 2016). The combination of these 2 predictive values, plus the confirmation of elevated levels of miRNA-122 in the total plasma of our patients, ensured us we had a patient population with exclusively early fibrotic patients. A third limitation is the fact that we only look at six miRNAs in the current study. With this study we wanted to investigate whether miRNAs that are related to HSC activation, could be detected in total plasma or circulating ECVs from patients with early stage liver fibrosis. Unbiased RNA sequencing of HSC-derived ECVs could identify other potential disease-specific biomarkers. This study also does not implicate that vesicle-associated miRNAs are a better biomarker than total plasma miRNA, but suggests that the use of vesicle-associated miRNAs indeed could give a more sensitive representation of the status of specific cell types, in this case the activation status of HSCs. Circulating ECVs could be a significant addition to current non-invasive biomarker panels to become even more sensitive for fibrosis progression, and for detection of early and small changes in fibrosis resolution. The former serves to identify patients at risk to develop advanced fibrosis, while the latter would be of great value for studying anti-fibrotic effects of drugs: if small changes could be more easily detected, the duration of clinical trials could be shortened.

## Conclusion

We found a significant difference in the miRNA expression profiles of total plasma and circulating ECVs from early fibrotic HBV/HCV-infected patients. Our results suggest the use of ECV-associated miRNAs as markers for the presence or absence of activated HSCs and thus early stage liver fibrosis.

## Author Contributions

JL study concept and design; acquisition of data; analysis and interpretation of data; statistical analysis; drafting of the manuscript; analysis. PJ acquisition of data; analysis and interpretation of data. SV analysis and interpretation of data; HR provision of samples; interpretation of data and critical revision of the manuscript. IM study concept and design; interpretation of data; critical revision of the manuscript. LvG study concept and design; obtained funding; interpretation of data; critical revision of the manuscript.

## Conflict of Interest Statement

The authors declare that the research was conducted in the absence of any commercial or financial relationships that could be construed as a potential conflict of interest.

## Footnotes

1 http://powerandsamplesize.com/Calculators/
