# Supplementary material for: Circulating ECV-Associated miRNAs as Potential Clinical Biomarkers in Early Stage HBV and HCV Induced Liver Fibrosis
Source: Front Pharmacol. 2017 Feb 9;8:56. doi: 10.3389/fphar.2017.00056 (PMC5298975; doi:10.3389/fphar.2017.00056)
Supplement: Supplementary file 1 [file Table_1.DOCX]

Supplementary Material

Circulating ECV-associated miRNAs as potential clinical biomarkers in early stage HBV and HCV induced liver fibrosis

Joeri Lambrecht^1^, Pieter Jan Poortmans^1^, Stefaan Verhulst^1^, Hendrik Reynaert^1,2^, Inge Mannaerts^1§^, Leo A. van Grunsven^1§^*

1. Department of Basic Biomedical Sciences, Liver Cell Biology Lab, Vrije Universiteit Brussel, Laarbeeklaan 103, 1090 Brussels, Belgium
2. Department of Gastro-enterology and Hepatology, UZ Brussel, Brussels, Belgium

***Correspondence:**

Leo A. van Grunsven

[Leo.van.grunsven@vub.ac.be](mailto:Leo.van.grunsven@vub.ac.be)

^§^ These authors have contributed equally to this work

## Supplementary table

**Supplementary table 1. Presumed targets of ECV-associated miRNAs.** TargetScan was addressed for identification of miRNA-pairs that were potentially involved in various pathways that are linked to HSC activation, being cell migration, cell proliferation, organization of extracellular matrix and the transforming growth factor beta (TGFβ) receptor pathway

|  | **Cell migration** | **Extracellular matrix organization** | **Cell proliferation** | **Transforming growth factor beta (TGFβ) receptor signaling pathway** |
| --- | --- | --- | --- | --- |
| MiRNA-92a | *WWC1, COL5A1, GPC6, ABI2, PRPF40A, CDC42BPA, SLC9A1, S1PR1, TGFB2, PALLD, SDC2, ITGAV, PTEN, MYO18A, SDC3, ABL2, RPS6KB1, PIK3CB, SNAI1, CUL3, PTPRK* | *FBN2, SPOCK2, COL5A1, NPNT, COL27A1, COL19A1, DAG1, FGB, HAPLN1, DMP1, IBSP, ITGAV, ITGA8, ITGA6, ATP7A, COL1A2, ATXN1L, ACAN, ITGA5, NF1, FBN1* | *SGK3, RBFOX2, KCNH1, NKX2-3, ABL2, EZH2, BMPR2, MBD2, PDS5B, TFAP2E* | *SMAD7, DKK3, BCL9, TRIM33, SOX11* |
| MiRNA-150 | *ERBB4, LAMC1, CDC42BPB, FUT8* | *DDR2, SPOCK2, LAMC1* | *TP53, ERBB4, GRHL2, KAT2A, CDC25A, BHLHE41, FGF1* | *CREB1, CBL, FUT8* |
| MiRNA-200b | *FSCN1, SHROOM2, GPC6, PLCG1, ABI2, FYN, AVL9, SDC2, PTEN, ERBB4, RHOA, HOXA5, LAMC1, EFNA1, RPS6KB1, PIK3CB, FLT1, PAK7, PDPK1, ERG, NANOS1* | *COL4A3, NPNT, FOXF2, FOXF1, LOX, DAG1, FN1, KDR, RECK, COL9A3, LAMC1, ATXN1L, NDNF, MFAP5* | *CKLF, ARHGEF1, LHX9, COL4A3BP, DACH1, COPS2, BHLHE41, YME1L1, KLF10, CITED2, ZEB1, LRP1, PAK7, RAC1, PDS5B, VTI1B, ERG, FSCN1, CDC27, DDIT4, EPS8, COL4A3, GAB1, KHDRBS1, EVI5, MAPRE1, PTEN, ERBB4, SIX1, WDR12, CUL5* | *CREB1, SMURF1, FERMT2, RHOA, SMAD9, CBL, JUN, SMAD2, SMAD7, KLF10* |
| MiRNA-192 | *COL5A1, S1PR1* | *COL5A1* | *H3F3B, TYMS, NR6A1, UBE2V2, ZFP36L* | *MTMR4, CBL* |
