# Supplementary material for: Circulating ECV-Associated miRNAs as Potential Clinical Biomarkers in Early Stage HBV and HCV Induced Liver Fibrosis
Source: Front Pharmacol. 2017 Feb 9;8:56. doi: 10.3389/fphar.2017.00056 (PMC5298975; doi:10.3389/fphar.2017.00056)
Supplement: Supplementary file 2 [file Data_Sheet_1.DOCX]

Supplementary Material

Circulating ECV-associated miRNAs as potential clinical biomarkers in early stage HBV and HCV induced liver fibrosis

Joeri Lambrecht^1^, Pieter Jan Poortmans^1^, Stefaan Verhulst^1^, Hendrik Reynaert^1,2^, Inge Mannaerts^1§^, Leo A. van Grunsven^1§^*

1. Department of Basic Biomedical Sciences, Liver Cell Biology Lab, Vrije Universiteit Brussel, Laarbeeklaan 103, 1090 Brussels, Belgium
2. Department of Gastro-enterology and Hepatology, UZ Brussel, Brussels, Belgium

***Correspondence:**

Leo A. van Grunsven

[Leo.van.grunsven@vub.ac.be](mailto:Leo.van.grunsven@vub.ac.be)

^§^ These authors have contributed equally to this work

## Supplementary Figure


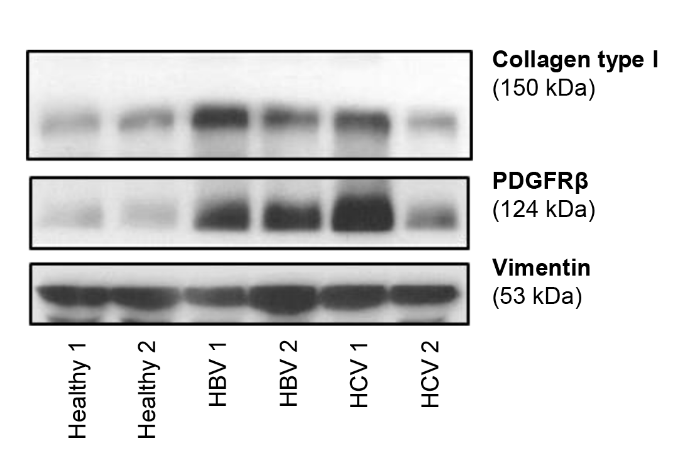


**Supplementary Figure 1:** **Detection of HSC-enriched proteins in lysates of plasma ECVs from early-stage HBV-and HCV-induced fibrotic subjects and healthy controls.** ECVs extracted from plasma obtained from F0/F1/F2 patients with chronic HBV and HCV infection, and a healthy control group were analyzed by western blot for expression of collagen type I and platelet-derived growth factor receptor beta (PDGFRβ), both markers associated with activating HSCs, and vimentin, which is a HSC-marker whose expression is not influenced by the activation status of the HSC.
